# Ensemble of Deep Learning Based Clinical Decision Support System for Chronic Kidney Disease Diagnosis in Medical Internet of Things Environment

**DOI:** 10.1155/2021/4931450

**Published:** 2021-12-27

**Authors:** Suliman A. Alsuhibany, Sayed Abdel-Khalek, Ali Algarni, Aisha Fayomi, Deepak Gupta, Vinay Kumar, Romany F. Mansour

**Affiliations:** ^1^Department of Computer Science, College of Computer, Qassim University, Buraydah 51452, Saudi Arabia; ^2^Department of Mathematics, Faculty of Science, Taif University, Taif, Saudi Arabia; ^3^Department of Mathematics, Faculty of Science, Sohag University, Sohag, Egypt; ^4^Department of Statistics, Faculty of Science, King Abdulaziz University, Jeddah, Saudi Arabia; ^5^Department of Computer Science & Engineering, Maharaja Agrasen Institute of Technology, Delhi, India; ^6^Department of Computer Engineering and Application, GLA University, Mathura, Uttar Pradesh, India; ^7^Department of Mathematics, Faculty of Science, New Valley University, El-Kharga 72511, Egypt

## Abstract

Recently, Internet of Things (IoT) and cloud computing environments become commonly employed in several healthcare applications by the integration of monitoring things such as sensors and medical gadgets for observing remote patients. For availing of improved healthcare services, the huge count of data generated by IoT gadgets from the medicinal field can be investigated in the CC environment rather than relying on limited processing and storage resources. At the same time, earlier identification of chronic kidney disease (CKD) becomes essential to reduce the mortality rate significantly. This study develops an ensemble of deep learning based clinical decision support systems (EDL-CDSS) for CKD diagnosis in the IoT environment. The goal of the EDL-CDSS technique is to detect and classify different stages of CKD using the medical data collected by IoT devices and benchmark repositories. In addition, the EDL-CDSS technique involves the design of Adaptive Synthetic (ADASYN) technique for outlier detection process. Moreover, an ensemble of three models, namely, deep belief network (DBN), kernel extreme learning machine (KELM), and convolutional neural network with gated recurrent unit (CNN-GRU), are performed. Finally, quasi-oppositional butterfly optimization algorithm (QOBOA) is used for the hyperparameter tuning of the DBN and CNN-GRU models. A wide range of simulations was carried out and the outcomes are studied in terms of distinct measures. A brief outcomes analysis highlighted the supremacy of the EDL-CDSS technique on exiting approaches.

## 1. Introduction

Internet of Things (IoT) aim is to interconnect and develop the connected things by computer network. Instead of utilizing higher energy consumption gadgets like phones, tabs, and machines [1], currently, some objects like air conditioners and room fresh units are computed by microcontroller utilizing sensing devices and provide the experimental result almost embedded in regular devices. IoT integrated with cloud computing (CC) method is highly beneficial while developing applications. A monitoring technique can be developed by incorporating cloud and IoT to monitor the infected people even if they are at distance that is highly employed by medical examiners [2]. Generally, IoT techniques keep on using cloud platforms for enhancing the efficacy in terms of data storage, programming abilities, computing, and utilization of resources efficiently. As well, cloud computing (CC) system attains superior experience from IoT by extending range to manage real time and provide distinct services dynamically in nature. Integration of cloud platform and IoT serves higher efficiency when compared to other cloud based methods. Some regions where this incorporation is employed are armed forces, hospitality, banking sectors, and home appliances. Among different applications, healthcare and medicinal are some of the stimulating research which assists rapid growth in medicinal and sensing gadgets [3].

CC aimed at utilizing IoT devices to transmit patient data (unstructured, structured, and semistructured healthcare information) on cloud platforms for configuring big data of patients [4]. Consequently, CC technique in medical services assists stakeholders in managing patient medical records, retrieving big data of patients, application of disease predictions, telemedicine, etc. [5]. Recently, the entire world faces difficulties of public health problems of chronic diseases like CKD which subsequently increases healthcare costs [6]. Based on threats and increases in the costs of CKD treatment, particularly in developing nations, earlier CKD predictions become a major challenge for healthcare centres and physicians in this country. Currently, network technologies and biomedical sensors offer a wide-ranging development in the fields of IoT [7], as a scheme where the smart healthcare device with unique identifiers could be communicated and connected for receiving and sending essential healthcare multimedia data to earlier detection in severe conditions, for example, threatening chronic disease such as CKD [8].

For effective and accurate rereading of the patient's healthcare state, each essential healthcare parameter and data gathered by IoT sensor could be analyzed via ML methods in prediction model which is demonstrated as an efficient solution in earlier medical detection. Data mining approaches like classification approaches as effective tool is extensively employed in anomaly detection and disease prediction in extensive research as an efficient method [9]. Based on the developments of utilizing current IoT devices and biomedical sensors, several health tracking and smart medical care schemes were introduced [10]. Mostly, the present research aims at earlier detection of some chronic diseases including diabetes mellitus, heart disease, and CKD where several factors influencing chronic disease have been used. But, considering each essential characteristic needed for predicting diseases, performance of prediction method, and the execution time still remains a challenge [11].

This study presents an ensemble of deep learning based clinical decision support systems (EDL-CDSS) for CKD diagnosis in the IoT environment. The EDL-CDSS technique involves the design of Adaptive Synthetic (ADASYN) technique for outlier detection process. In addition, an ensemble of three models such as deep belief network (DBN), kernel extreme learning machine (KELM), and convolutional neural network with gated recurrent unit (CNN-GRU) takes place. Besides, quasi-oppositional butterfly optimization algorithm (QOBOA) is used for the hyperparameter tuning of the DBN and CNN-GRU models. To examine the improved CKD detection outcomes of the EDL-CDSS technique, an extensive experimental analysis is carried out.

## 2. Literature Review

This section offers a brief survey of recently developed CKD classification models. Arulanthu and Perumal [12] presented an optimum cloud and IoT based DSS for CKD detection. The presented technique utilizes SA based FS method with RMSProp Optimizer based LR method named SA-RMSPO-LR to categorize the presence of CKD from healthcare information. Noor et al. [13] introduced a primary healthcare scheme for the treatments of Chronic Renal Disease/CKD patient with the idea of IoT that consist of the nutrition of food mostly focusing on the levels of salt intake, patient log, activity level, water intake, monitoring the sleep pattern, etc. for providing necessary improvement required for the advancement of their healthcare status.

Arulanthu and Perumal [14] introduced an online medical decision support system (OMDSS) for predicting CKD. The proposed algorithm includes a group of phases, i.e., classification, data gathering, and preprocessing of healthcare information for CKD prediction. Additionally, the parameters of adaptive learning rate optimization, LR, and Adaptive Moment Estimation (Adam) methods were employed. Bhaskar and Manikandan [15] presented a novel sensing method for the automatic diagnosis of CKD. The salivary urea concentration is observed for detecting the diseases. A novel sensing method is presented for monitoring the urea stages in the saliva samples. Moreover, analyzing the raw signals attained from the sensor, they have executed a 1D-DL-CNN method that is integrated by an SVM classification. Abdelaziz et al. [16] designed a hybrid intelligent method for CKD prediction based cloud-IoT by utilizing 2 smart technologies that are NN and LR. NN is employed for predicting CKD. LR is utilized for determining crucial factors that influenced CKD.

Ma et al. [17] proposed a Heterogeneous Modified Artificial Neural Network (HMANN) for earlier segmentation, diagnosis, and detection of CKD failure on the IoMT environment. Moreover, the presented method is categorized as an SVM and MLP with a BP method. In Hosseinzadeh et al. [18], a diagnostic predictive method for CKD and its seriousness is presented which employs IoT multimedia dataset. Because the factors affecting CKD are very large and the amount of IoT multimedia information is generally large, choosing dissimilar features based on physician's medical experience and observation as well as earlier researches for CKD in various types of multimedia datasets is performed for assessing the performance measure of CKD predictions and its determination level through dissimilar classification methods.

## 3. The Proposed Model

**Figure 1:**
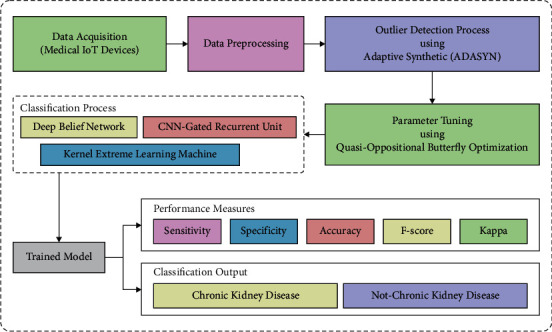
Overall process of EDL-CDSS technique.

The common structure of the presented method has been demonstrated in Figure 1. The major component in the presented method is benchmark CKD dataset, IoT devices embedded in the patients, patient healthcare records, cloud database server (CDS), security system, data collection module, disease prediction module, and CKD diagnosis. The wearable IoT healthcare devices are considered IoT devices that are located in the person's body. The standard CKD datasets from the UCI repository are employed. The healthcare datasets contain the previous information of the patient details that are collected from the clinics. This dataset is stored in the CDS. The security system recollects the information from the data collecting model. This information would be stored in a private way by using different stages of format conversion, information retrieval, and data integration. The secure information would be again stored in the CDS and it would be retrieved when it is needed. The disease prediction module and CKD diagnosis employ ensemble DL classifiers for the detection of CKD. In the training stage, the medical information from the CKD dataset and patient healthcare records are utilized for training the EDL-CDSS technique.

### 3.1. Data Acquisition

The presented architecture has 3 different types of information. In this phase, the medical information of the patients would be collected by using wearable IoT devices that are working by sensor nodes. The wearable devices are located on the patient's body to gather the certain patient's medical data frequently in a certain time period. Generally, the IoT devices in the human body check each sensed medical information either it is normal or not. The presented method uses 4G mobile network for transferring the sensed medical information to the CDS. Furthermore, the CKD dataset from UCI repository is applied for mapping the actual data that is created using the IoT gadgets. In addition, patients' healthcare details are applied for mapping the real information created using the individual patient information.

### 3.2. Data Preprocessing

For providing effectual efficiency with minimum cost to data mining procedures, the quality of data is optimum. The value missing from the database has been filled under the entire CKD datasets. In few cases, if the continuous features exist, these techniques are synchronized for building discrete traits. It has any noisy and missing values from all samples. In order to improve the performance of medicinal information, a novel data was preprocessed.

### 3.3. ADASYN Based Outlier Detection

In this study, ADASYN is used to remove outliers, which is an extension of SMOTE. ADASYN has been found to be useful in medical imaging applications for detection of premature delivery, retinal health diagnosis, and diagnosis of focal liver lesions. Even though ADASYN is dependent on SMOTE, compared to borderline-SMOTE, ADASYN generates distinct synthetic instances for the minority class based on its distribution and for the borderline samples. Besides that, SMOTE offers equal opportunity for all the minority instances to get elected while in ADASYN the selection procedure is depending on the minority class distribution. The synthetic instance is generated according to the majority nearest neighbours through the KNN process. The method employs weighted dissemination for different minority class samples as per their complexity levels in training [19]. It generates additional synthetic instances for samples from minority class that is strenuous for training compared to those cases which is easy to train. The method initiates by evaluating the level of class imbalance. Then, it evaluates the amount of synthetic instances that need to be generated for the minority class by detecting KNN as per the Euclidean distance in n-dimension space. According to the ratio of density spread, the technique estimates the amount of synthetic data instances needed to be generated for the minority class.

### 3.4. Ensemble Classification

At this stage, the outliers removed data are fed into the ensemble classification model, which comprises three models, namely, KELM, DBN, and CNN-GRU. These three feature vectors can be defined as follows:

(1) $$\begin{array}{c} f_{\text{KELM}1\times n}=\left\{{\text{KELM}}_{1\times 1},{\text{KELM}}_{1\times 2},{\text{KELM}}_{1\times 3},\ldots ,{\text{KELM}}_{1\times n}\right\},\\ f_{\text{DBN}1\times m}=\left\{{\text{DBN}}_{1\times 1},{\text{DBN}}_{1\times 2},{\text{DBN}}_{1\times 3},\ldots ,{\text{DBN}}_{1\times m}\right\},\\ f_{\text{CNN}-\text{GRU}1\times l}=\left\{\text{CNN}-{\text{GRU}}_{1\times 1},\text{CNN}-\text{GRUCNN}-{\text{GRU}}_{1\times 2},\text{CNN}-{\text{GRU}}_{1\times 3},\ldots ,\text{CNN}-{\text{GRU}}_{1\times l}\right\}. \end{array}$$

In addition, the derived individual features are combined into a single vector, using the following equation:

(2) $$\begin{array}{c} \text{Fused}{\left(\text{features vector}\right)}_{1\times q}=\overset{3}{\underset{i=1}{\sum }}f_{\text{KELM}1\times n},f_{\text{DBN}1\times m},\;f_{\text{CNN}-\text{GRU}1\times l}, \end{array}$$

where *f* represents fused vectors (1 × 1186).

#### 3.4.1. KELM Model

The output function of ELM in case of one output node is

(3) $$\begin{array}{c} f\left(x\right)=\sum_{i=1}^{L}\beta_{i}G\left(a_{i},\;b_{i},\;x\right)=\beta \cdot h\left(x\right), \end{array}$$

where *β*=[*β*_1_,   …, *β*_*L*_]^*T*^ denotes the output weight vector. *G*(*a*_*i*_,  *b*_*i*_,  *x*) indicates the output of ith hidden layer, as well as the node variable, is arbitrarily created. *h*(*x*)=[*G*(*a*_1_,  *b*_1_,  *x*),   …, *G*(*a*_*L*_,  *b*_*L*_,  *x*)]^*T*^ represent the output vector of hidden layer in relation to the input. Afterwards the kernel function can be determined by

(4) $$\begin{array}{c} \Omega_{\text{ELM}}=HH^{T}:\;\Omega_{\text{ELM}}=h\left(x_{i}\right)\cdot h\left(x_{j}\right)=K\left(x_{i},\;x_{j}\right). \end{array}$$

The output function of ELM classifiers is expressed by

(5) $$\begin{array}{c} f\left(x\right)=h\left(x\right)H^{T}{\left(\frac{I}{\lambda }+HH^{T}\right)}^{-1}T\\ =\left[\begin{array}{c} K\left(x,x_{1}\right)\\ \vdots \\ K\left(x,x_{N}\right) \end{array}\right]{\left(\frac{I}{\lambda }+\Omega_{\text{ELM}}\right)}^{-1}T, \end{array}$$

in which *I* represents the identity matrix, *λ* indicates the normalization coefficient, and *T* signifies the trained set label [20]. Afterwards using this model, we do not want to know the certain form of the feature map *h*(*x*) but utilize the kernel function to resultant computation. Therefore, the arbitrarily generated bias and weights are evaded, and it is not necessary to set the amount of hidden layer *L*.

#### 3.4.2. DBN Model

Since Hinton was developed in 2006, the DBN deployment is made up of stacked RBM. First, the network employs the Contrastive Divergence (CD) method to unsupervised training of stacked RBM, later applying the BP method for fine-tuning the node parameter in the whole DBN network. Mainly, DBN training consists of fine-tuning and pretraining. The pretraining phase employs all the layers of RBM to implement unsupervised training on unlabeled sample data and simultaneously applies the CD method for tuning all the layers of RBM parameter. Afterwards, the pretraining DBN estimates the network error of all the layers by using the BP method and adjusts the parameters of all the layers by using BP method, for realizing the global fine-tuning of the node weight of the whole DBN network. The undirected graph method consists of hidden layer and visual layer. All the layers have multiple nodes. RBM is an energy based method, and energy function can be determined as hidden layer *h*=(*h*_*j*_)_*m*_ and visible layer *v*=(*v*_*i*_)_*n*_:

(6) $$\begin{array}{c} E_{\theta }\left(v,\;h\right)=-\sum_{i=0}^{n_{v}}a_{i}v_{i}-\sum_{j=0}^{n_{h}}b_{j}h_{j}-\sum_{i=0}^{n_{v}}\sum_{j=0}^{n_{h}}v_{i}w_{ij}h_{j}, \end{array}$$

where *a*_*i*_ represents the bias of *i*th neurons from visible layer and *b*_*j*_ indicates the bias of *j*th neurons under the hidden layer; *θ*=[*w*=(*w*_*ij*_)_*n*×*m*_,  *a*=(*a*_*i*_)_*n*_,  *b*=(*b*_*j*_)_*m*_] signifies the parameter of RBM method; *w*_*ij*_ shows the connection weight among the hidden layer *h*_*j*_ and visible layer *v*_*i*_; *nh* denotes the amount of hidden layers and *n*_*v*_ is the amount of visible layers.

(7) $$\begin{array}{c} P_{\theta }\left(v,\;h\right)=\frac{1}{Z_{\theta }}\;\exp\;\left(-E_{\theta }\left(v,\;h\right)\right). \end{array}$$

Among others, *Z*_*θ*_ represents the standardization factor as follows:

(8) $$\begin{array}{c} Z_{\theta }=\sum_{v}\sum_{h}\exp\left(-E_{\theta }\left(v,\;h\right)\right). \end{array}$$

Once the *v* state of neurons over the visible layer is provided [21], the probabilities of jth neurons *h*_*j*_ from the hidden layer was initiated (by the probability 1) as

(9) $$\begin{array}{c} P_{\theta }\left(h_{j}=1|v\right)=\text{sigmoid }\left(b_{j}+\sum_{i=0}^{n_{v}}w_{ij}v_{i}\right). \end{array}$$

Once the *h* state of neuron on hidden layer is provided, the probability that *i*th neuron *v*_*i*_ from the visible layer has initiated (by the probability 1) is

(10) $$\begin{array}{c} P_{\theta }\left(v_{i}=1|h\right)=\text{sigmoid }\left(a_{i}+\sum_{j=0}^{n_{v}}w_{ji}h_{j}\right), \end{array}$$

in which, sigmoid(*x*)=(1+exp(−*x*))^−1^ represent the activation function, whereas *x* is in the range of zero and one.

#### 3.4.3. CNN-GRU Model

CNN is a multilayer neural network that is made up of multiple pooling, fully connected, and convolution layers, with the pooling and convolution layers. Furthermore, the FC layers are employed for classifying the CKD from the extracted feature map. All the layer inputs are interconnected to preceding layer output, and the outputs pass to following layer. The parameter of the networks is trained and shared by BP technique. The LSTM consists of update, input, and forget gates. In the meantime, the GRU is simple when compared to the LSTM because it has only 2 gates that are updated and reset gates. This gate is employed for determining either the data is beneficial or not. In GRU, *W*_*Z*_, *W*_*r*_, and *W* imply the update gate, reset gate, and candidate data, correspondingly. Besides being fully connected with the neuron in the preceding hidden neuron at time *t*, all the neurons from GRU hidden layers are FC for each neuron under the present hidden layer at time *t* − 1. The *l*^th^ hidden layer output is calculated as follows:

(11) $$\begin{array}{c} h_{t}^{l}=\left(1-z_{t}\right)*h_{t-1}^{l}+z_{t}*h_{t}^{-l}, \end{array}$$

where *z*_*t*_ represents an update gate, and *h*_*t*_^−*l*^ indicates the candidate memory data. Furthermore, *z*_*t*_ is provided by equation (12) which controls how many data of the preceding and present memory would be added/forgotten.

(12) $$\begin{array}{c} z_{t}=\text{sigmoid}\left(W_{Z}\cdot \left[h_{t-1}^{l},\;h_{t}^{l-1}\right]\right). \end{array}$$

The candidate data value *h*_*t*_^−*l*^ is estimated as

(13) $$\begin{array}{c} h_{t}^{-l}=\;\tanh\;\left(W\cdot \left[r_{t}*h_{t-1}^{l},h_{t}^{l-1}\right]\right), \end{array}$$

in which *r*_*t*_ determined by equation (15) is the GRU reset gate that effectively reset the data in the memory [22].

(14) $$\begin{array}{c} r_{t}=\text{sigmoid}\left(W_{r}\cdot \left[h_{t-1}^{l},\;h_{t}^{l-1}\right]\right). \end{array}$$

Sigmoid and  tanh  represent activating functions in the following:

(15) $$\begin{array}{c} \text{sigmoid }\left(x\right)=\frac{1}{1+e^{-x}},\\ \tanh\;\left(x\right)=\frac{e^{x}-e^{-x}}{e^{x}+e^{-x}}. \end{array}$$

Compared to the CNN-FC layer, the estimated data by the GRU comprises historical state, wherein the neuron value at time *t* can be described by the information in the preceding layer at time *t*, and it is defined by the information stored in the GRU cell at time *t* l (equation (11)).

**Figure 2:**
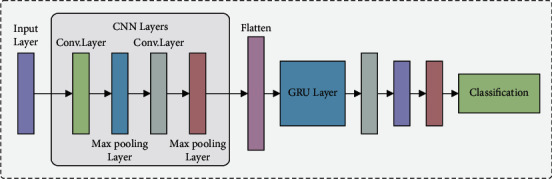
Framework of CNN-GRU model.

The GRU network replaces CNN-FC layer to convert the classification process as to sequential task, where the classification result of all the feature maps is included in the following feature map classification in the similar hidden layer for improving the CNN detection accuracy. The presented CNN-GRU framework has been demonstrated in Figure 2. In CNN-GRU architecture, the pooling, original CNN input, and convolution layers parameters with sizes are not changed to implement the features extraction. All the output feature maps of the *l*^th^ convolution layer are calculated by

(16) $$\begin{array}{c} O_{-}{\text{conv}}_{j}^{l}=\text{sigmoid}\left(\sum_{i=1}^{M}\text{convn}\left(a_{i}^{l-1*}k_{ij}^{l}\right)+b_{j}^{l}\right), \end{array}$$

in which *a*_*i*_^*l*−1^ represent the *i*th feature maps in the overall feature maps *M* of the *l* − 1^th^ preceding layer, and *k*_ij_^*l*^ represents the *l*^th^ layer kernel. The values at the location of (*m*, *n*) in the convolutional process are determined by

(17) $$\begin{array}{c} \text{convn }\left(a_{i}^{l-1},k_{ij}^{l}\right)\left[m\right]\left[n\right]=\sum_{w=0}^{k-1}\sum_{l=0}^{k-1}k_{ij}^{l}\left[w\right]{\left[l\right]}^{*}a_{i}^{l-1}\left[m+w\right]\left[n+l\right], \end{array}$$

where *k*=5 denotes the convolution kernel width. During pooling layer, all the output feature map values at the location of (*m*,  *n*) are estimated by utilizing average pooling technique as follows, in which *a*_*j*_ indicates the *j*^th^ feature map of *l* − 1 convolution layer, and *K*=2 denotes the pooling kernel width:

(18) $$\begin{array}{c} {\text{aver}}_{-}{\text{pool}}_{j}^{l}\left[m\right]\left[n\right]=\frac{1}{4}\sum_{l,w=0}^{K-1}a_{j}^{l-1}\left(m+l,\;n+w\right). \end{array}$$

Next, each feature of all the feature maps in the last CNN pooling layers is interconnected to a respective GRU method. This implies that there are overall twelve GRU networks, and all the GRU network sets 3 layers, involving an output neuron of ten layers, an input neuron of twenty-five layers, and a hidden neuron of fifty layers. Lastly, the GRU output is activated using softmax function determined in equation (19) to categorize the CKD. In the testing stages, each GRU output has been chosen for finding the final detection outcome.

(19) $$\begin{array}{c} y_{k}=\frac{\text{exp}\left(w_{k}h\right)}{\sum_{k},\;\exp\left(w_{k}\cdot h\right)}. \end{array}$$

### 3.5. QOBOA Based Hyperparameter Optimization

To optimally tune the hyperparameter involved in the DBN and CNN-GRU models, the QOBOA is utilized and thereby boosts the overall CKD classification performance. BOA [23] is a population based nature simulated optimization method, depending on the food hunting system of butterflies. Once butterflies move from one place to another, it releases a fragrance with intensity, i.e., transferred over the distance. The other butterflies could find this fragrance and be attracted to it according to the intensity level of fragrance. Once the butterfly senses the optimal butterfly fragrance it begins to move toward it. This procedure is called a local search. It produces fragrance with intensity once it begins to move. Another butterfly was attracted to it as per its level of fragrance. It can be determined by

(20) $$\begin{array}{c} f=c\times I^{a}, \end{array}$$

where *c* represents a sensor modality, *I* indicates the fragrance level, and *a* denotes the degree of fragrance absorptions. The 2 main phases of the process are given in the following. Every butterfly releases fragrance once it starts moving and another butterfly is attracted to it as per its level of intensity of fragrance. This procedure is described in the following:

(21) $$\begin{array}{c} x_{i}\left(t+1\right)=x_{i}\left(t\right)+\left(\alpha^{2}\times g^{*}-x_{i}\left(t\right)\right)\times f_{i}. \end{array}$$

While *x*_*i*_(*t*) indicates a vector that denotes the butterfly (solution) at iteration *t*, *g*^*∗*^ represents the total optimal solution, *α* shows an arbitrary value within [0,1], and *f*_*i*_ denotes a fragrance of *j*^th^ butterfly. Once the butterfly fails to find the fragrance of another butterfly, it could arbitrarily move in the searching space. The procedure is described in the following:

(22) $$\begin{array}{c} x_{i}\left(t+1\right)=x_{i}\left(t\right)+\left(\alpha^{2}\times x_{j}\left(t\right)-x_{k}\left(t\right)\right)\times f_{i}. \end{array}$$

In the equation, *x*_*j*_(*t*), *x*_*k*_(*t*) indicate 2 vectors that show two distinct butterflies in a similar population. A *p* switching probability was utilized in BOA for switching from general global search for intensive local search.

For improving the efficiency of the model based on optimal solution and convergence the concept of opposition based learning was presented in BOA [24]. A variables quasi‐opposite value of a candidate solution is randomly considered among the mirror point of the parameter and the midpoint of the searching space. It can be expressed in the following equation:

(23) $$\begin{array}{c} x_{i,j}^{q}=\text{rand}\left(a,b\right),\\ a=\frac{x_{i,j}^{\min}+x_{i,j}^{\max}}{2},\\ b=x_{i,j}^{\min}+x_{i,j}^{\max}-x_{i,j}. \end{array}$$

Here, *x*_*i*,*j*_ denotes the *j*^th^ dimension of *j*^th^ candidate solution; *x*_*i*,*j*_^min^, *x*_*i*,*j*_^max^ indicate the minimal and maximal values of *x*_*i*,*j*_, and *x*_*i*,*j*_^*q*^ represents quasi-opposite value of *x*_*i*,*j*_.

## 4. Results and Discussion

**Figure 3:**
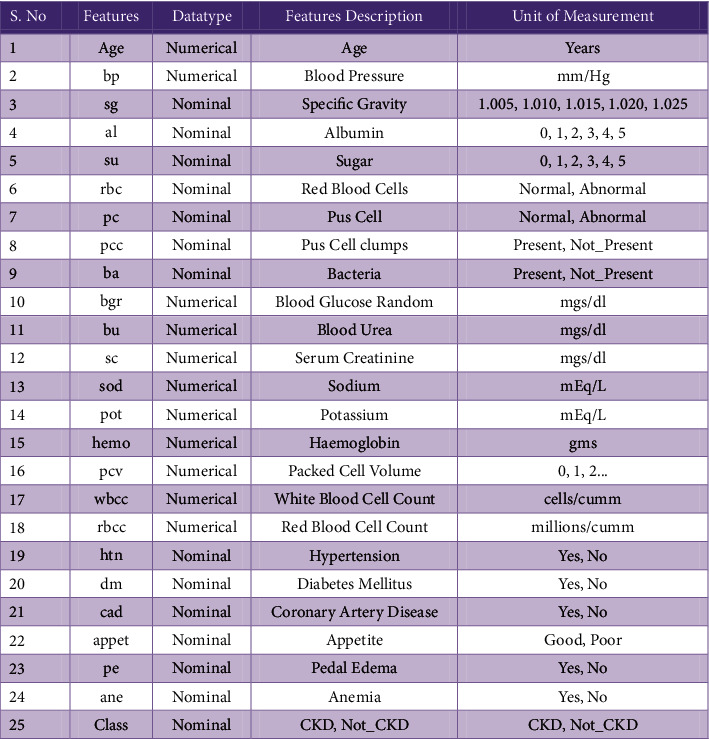
Attributes involved in CKD dataset.

The EDL-CDSS technique is simulated using Python 3.6.5 tool and the results are examined using benchmark CKD dataset, which is publically available at https://archive.ics.uci.edu/ml/datasets/chronic_kidney_disease. The dataset includes 400 samples with 25 attributes. Among the available samples, 250 samples fall into CKD category and the residual of 150 samples come under Not-CKD category. The features involved in the CKD dataset are given in Figure 3.

**Figure 4:**
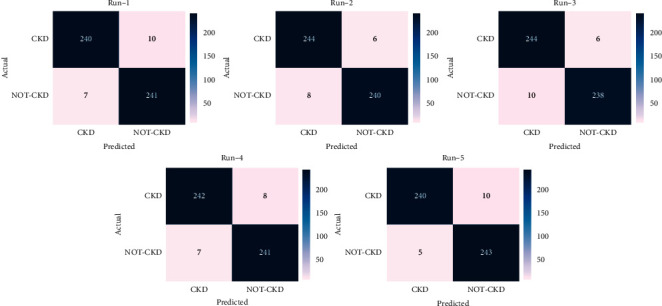
Confusion matrix analysis of EDL-CDSS technique with different runs.

Figure 4 shows the set of confusion matrices obtained by the EDL-CDSS technique on the test CKD data under five runs. The results show that the EDL-CDSS technique has classified the CKD and Not-CKD instances correctly. For instance, with run-1, the EDL-CDSS technique has identified 240 instances into CKD and 241 instances into Not-CKD. Meanwhile, with run-2, the EDL-CDSS manner has identified 244 instances into CKD and 240 instances into Not-CKD. Eventually, with run-3, the EDL-CDSS method has identified 244 instances into CKD and 238 instances into Not-CKD. Moreover, with run-4, the EDL-CDSS system has identified 242 instances into CKD and 241 instances into Not-CKD. Furthermore, with run-5, the EDL-CDSS manner has identified 240 instances into CKD and 243 instances into Not-CKD.

**Figure 5:**
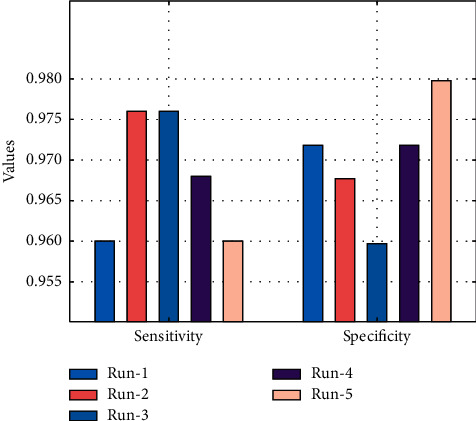
Sensitivity and specificity analysis of EDL-CDSS method with different runs.

Table 1 offers the overall CKD classification performance of the EDL-CDSS technique under five runs.  Figure 5 demonstrates the sens_*y*_ and spec_*y*_  analysis of the EDL-CDSS technique under five test runs. The figure reported that the EDL-CDSS technique has gained increased values of sens_*y*_ and spec_*y*_. For instance, with run-1, the EDL-CDSS technique has attained sens_*y*_ and spec_*y*_ of 0.9600 and 0.9718, respectively. Besides, with run-3, the EDL-CDSS technique has resulted in sens_*y*_ and spec_*y*_  of 0.9760 and 0.9597, respectively. Lastly, with run-5, the EDL-CDSS technique has accomplished sens_*y*_ and spec_*y*_ of 0.9600 and 0.9797, respectively.

**Figure 6:**
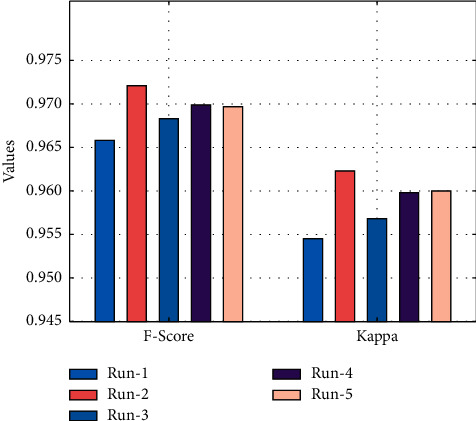
F-score and kappa analysis of EDL-CDSS method with different runs.

Figure 6 determines the *F*_score_ and kappa   analysis of the EDL-CDSS system under five test runs. The figure stated that the EDL-CDSS approach has reached improved values of *F*_score_ and kappa. For instance, with run-1, the EDL-CDSS technique has gained *F*_score_ and kappa of 0.9658 and 0.9545 correspondingly. Also, with run-3, the EDL-CDSS system has resulted in *F*_score_ and kappa of 0.9683 and 0.9568 correspondingly. At last, with run-5, the EDL-CDSS methodology has accomplished *F*_score_ and kappa of 0.9697 and 0.9600, respectively.

**Figure 7:**
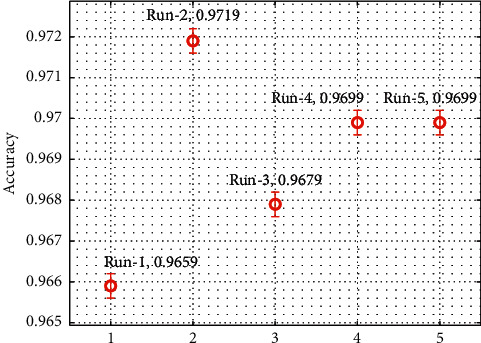
Accuracy analysis of EDL-CDSS method with different runs.

Figure 7 inspects the accu_*y*_ analysis of the EDL-CDSS technique on the test dataset. The results show that the EDL-CDSS technique has obtained improved CKD classification performance with the maximum accu_*y*_ of 0.9659, 0.9719, 0.9679, 0.9699, and 0.9699 under five test runs, respectively.

**Figure 8:**
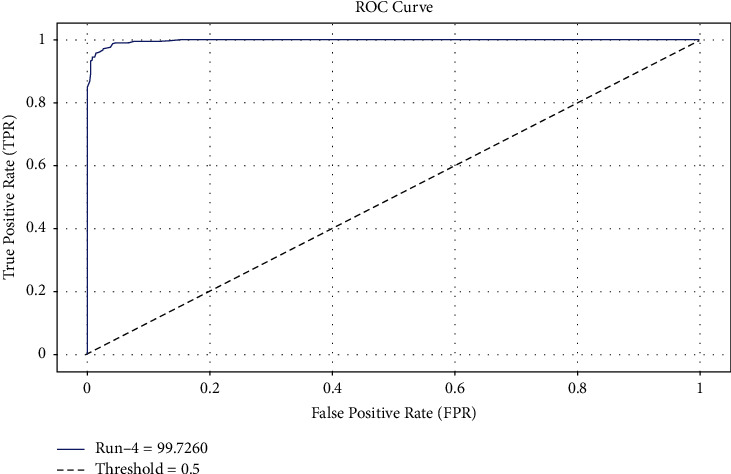
ROC analysis of EDL-CDSS method.

The ROC analysis of the EDL-CDSS technique on test dataset is demonstrated in Figure 8. The figure obviously shows that the EDL-CDSS manner has the ability to accomplish increased classification performance with the maximal ROC of 99.7260.

**Figure 9:**
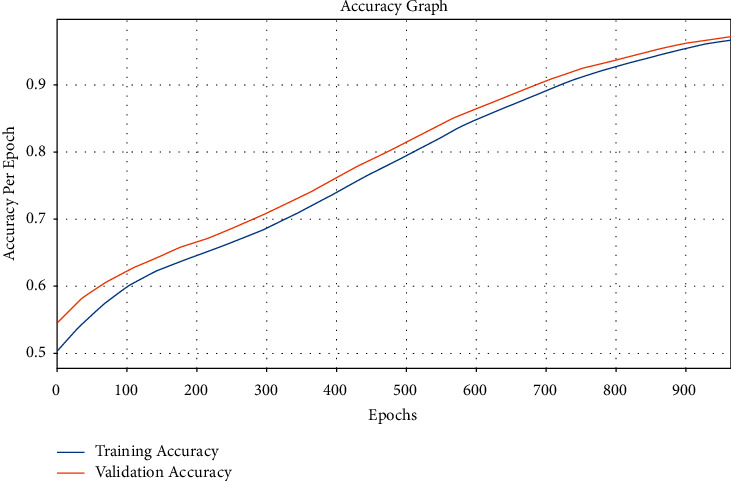
Accuracy graph analysis of EDL-CDSS method.

Figure 9 depicts the accuracy analysis of the EDL-CDSS method on test dataset. The outcomes displayed that the EDL-CDSS algorithm has accomplished improved performance with increased training and validation accuracy. It can be noticed that the EDL-CDSS technique has reached increased validation accuracy over the training accuracy.

**Figure 10:**
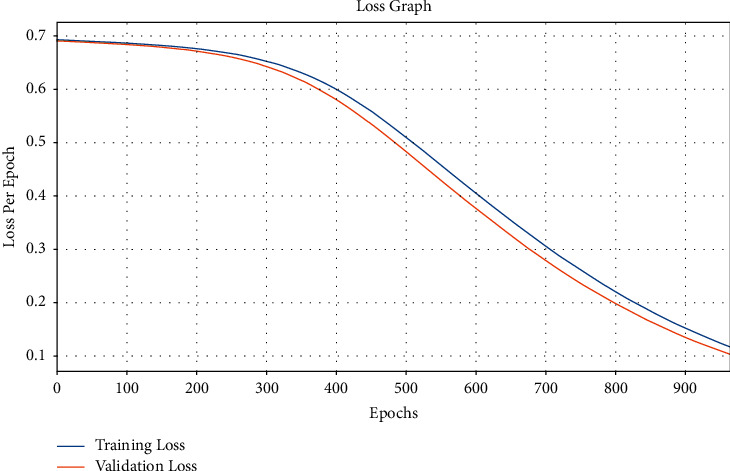
Loss graph analysis of EDL-CDSS method.

Figure 10 showcases the loss analysis of the EDL-CDSS approach on test dataset. The outcomes established that the EDL-CDSS system has resulted in a proficient outcome with the minimum training and validation loss. It can be stated that the EDL-CDSS system has offered lower validation loss over the training loss.

**Figure 11:**
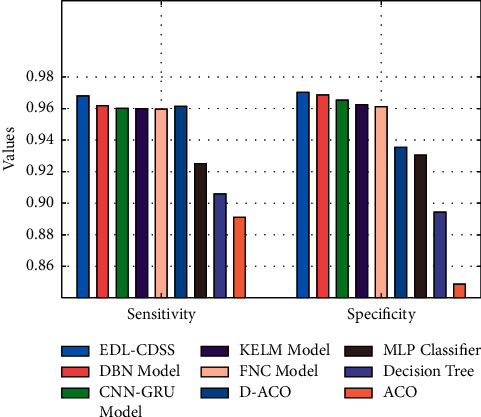
Comparative analysis of EDL-CDSS technique with existing methods.

Finally, a detailed comparative results analysis of the EDL-CDSS manner with recent methods takes place in Table 2 [25]. A brief comparative sens_*y*_ and spec_*y*_ analysis of the EDL-CDSS approach with existing ones is provided in Figure 11. The figure reported that the DT model has obtained least performance with the sens_*y*_ and spec_*y*_ of 0.9060 and 0.8944. Along with that, the MLP system has reached slightly enhanced outcome with the sens_*y*_ and spec_*y*_ of 0.9251 and 0.9305. In line with that, the ACO, FNC, KELM, CNN-GRU, and D-ACO models have obtained moderately closer CKD classification performance with nearer values of sens_*y*_ and spec_*y*_. Though the DBN model has resulted in near optimal outcome with the sens_*y*_ and spec_*y*_ of 0.9618 and 0.9686, the proposed EDL-CDSS method has depicted the other approaches with the superior sens_*y*_ and spec_*y*_ of 0.9680 and 0.9702.

**Figure 12:**
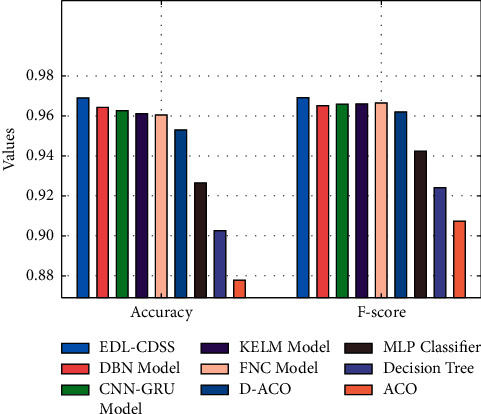
Accuracy and F-score analysis of EDL-CDSS technique with existing methods.

A detailed comparative acc_*y*_ and *F*_score_ analysis of the EDL-CDSS algorithm with existing ones is offered in Figure 12. The figure stated that the ACO approach has attained worse performance with the acc_*y*_ and *F*_score_ of 0.8778 and 0.9073. Besides, the DT technique has achieved somewhat increased results with the acc_*y*_ and *F*_score_ of 0.9026 and 0.9241. Likewise, the MLP, D-ACO, FNC, KELM, and CNN-GRU methodologies have reached moderately closer CKD classification performance with closer values of acc_*y*_ and *F*_score_. But the DBN technique has resulted in near optimum outcome with the acc_*y*_ and *F*_score_ of 0.9643 and 0.9651; the projected EDL-CDSS system has portrayed the other approaches with higher acc_*y*_ and *F*_score_ of 0.9691 and 0.9692.

After examining the above mentioned tables and figures, it can be ensured that the EDL-CDSS method has the capability of proficiently detecting the presence of CKD in the IoT environment. The enhanced performance of the proposed model is due to the inclusion of ensemble classification and QOBOA based hyperparameter tuning.

## 5. Conclusion

In this study, a novel EDL-CDSS approach was derived for CKD detection and classification in the IoT enabled cloud environment. The proposed EDL-CDSS technique encompasses distinct stages of operations, namely, data gathering preprocessing, ADASYN based outlier detection, ensemble classification, and QOBOA based hyperparameter tuning. For classification process, KELM, DBN, and CNN-GRU models are employed in which the hyperparameters of the DBN and CNN-GRU models are optimally tuned by the use of QOBOA. To examine the improved CKD detection outcomes of the EDL-CDSS approach, a huge range of simulations are implemented and the outcomes are studied with respect to various measures. A detailed comparative results analysis highlighted the supremacy of the EDL-CDSS manner on the existing approaches. Therefore, the proposed EDL-CDSS technique can be utilized as an effective tool for CKD diagnosis. In future, the proposed model can be applied to detect other diseases such as heart disease, diabetes, etc.

## Figures and Tables

**Table 1:** Result analysis of EDL-CDSS technique with five runs in terms of distinct measures.

No. of runs	Sensitivity	Specificity	Accuracy	F-score	Kappa
Run-1	0.9600	0.9718	0.9659	0.9658	0.9545
Run-2	0.9760	0.9677	0.9719	0.9721	0.9623
Run-3	0.9760	0.9597	0.9679	0.9683	0.9568
Run-4	0.9680	0.9718	0.9699	0.9699	0.9598
Run-5	0.9600	0.9798	0.9699	0.9697	0.9600
Average	**0.9680**	**0.9702**	**0.9691**	**0.9692**	**0.9587**

**Table 2:** Comparative analysis of various classifiers on CKD dataset.

Models	Performance measures			
	Sensitivity	Specificity	Accuracy	F-score
EDL-CDSS	0.9680	0.9702	0.9691	0.9692
DBN model	0.9618	0.9686	0.9643	0.9651
CNN-GRU model	0.9601	0.9653	0.9627	0.9659
KELM model	0.9599	0.9624	0.9611	0.9660
FNC model	0.9597	0.9612	0.9605	0.9665
D-ACO	0.9614	0.9354	0.9530	0.9620
MLP classifier	0.9251	0.9305	0.9265	0.9424
Decision tree	0.9060	0.8944	0.9026	0.9241
ACO	0.8910	0.8487	0.8778	0.9073

## Data Availability

The dataset used in this paper is publicly available via the following link: https://archive.ics.uci.edu/ml/datasets/chronic_kidney_disease.
